# Rationale for the Use of Upfront Whole Brain Irradiation in Patients with Brain Metastases from Breast Cancer

**DOI:** 10.3390/ijms15058138

**Published:** 2014-05-08

**Authors:** Agnes V. Tallet, David Azria, Emilie Le Rhun, Fabrice Barlesi, Antoine F. Carpentier, Antony Gonçalves, Sophie Taillibert, Frédéric Dhermain, Jean-Philippe Spano, Philippe Metellus

**Affiliations:** 1 Department of Radiation Oncology, Institut Paoli Calmettes, Marseille 13009, France; 2 Department of Radiation Oncology and INSERM U896, CRLC Val d’Aurelle, Montpellier 34000, France; E-Mail: David.Azria@icm.unicancer.fr; 3 Breast Unit, Department of Medical Oncology, Oscar Lambret Center, Lille Cedex 59020, France; E-Mail: le_rhun@yahoo.fr; 4 Multidisciplinary Oncology and Therapeutic Innovations Department & Centre Investigation Clinique, Aix Marseille University—Assistance Publique Hôpitaux de Marseille, Marseille 13011, France; E-Mail: Fabrice.BARLESI@ap-hm.fr; 5 Department of Neurology, Hopital Avicenne, Assistance Publique des Hopitaux de Paris, Bobigny 93000, France; E-Mail: antoine.carpentier@avc.aphp.fr; 6 Department of Medical Oncology, Institut Paoli Calmettes, Marseille 13009, France; E-Mail: goncalvesa@ipc.unicancer.fr; 7 Department of Radiation Oncology and Department of Neuro-Oncology, Pitié-Salpétrière Hospital, Paris 75013, France; E-Mail: sophie.taillibert@gmail.com; 8 Department of Radiation Oncology, Institut Gustave Roussy University Hospital, Villejuif 94805, France; E-Mail: Frederic.DHERMAIN@gustaveroussy.fr; 9 Department of Medical Oncology, GH Pitié-Salpêtrière, Université Paris 6, Paris 75013, France; E-Mail: jean-philippe.spano@psl.aphp.fr; 10 Department of Neurosurgery, Hopital La Timone, Assistance Publique-Hôpitaux de Marseille and INSERM UMR 911, Marseille 13006, France; E-Mail: Philippe.METELLUS@ap-hm.fr; 11 Groupe de Réflexion sur la Prise en Charge des MétAstases Cérébrales (GRPCMaC), Marseille 13009, France

**Keywords:** whole brain radiation therapy, breast cancer, brain metastases, controlled extra-cranial disease, overall survival

## Abstract

Breast cancer is the second most common cause of brain metastases and deserves particular attention in relation to current prolonged survival of patients with metastatic disease. Advances in both systemic therapies and brain local treatments (surgery and stereotactic radiosurgery) have led to a reappraisal of brain metastases management. With respect to this, the literature review presented here was conducted in an attempt to collect medical evidence-based data on the use of whole-brain radiotherapy for the treatment of brain metastases from breast cancer. In addition, this study discusses here the potential differences in outcomes between patients with brain metastases from breast cancer and those with brain metastases from other primary malignancies and the potential implications within a treatment strategy.

## 1. Introduction

In the last decade, the management of brain metastases (BM) has changed radically. Historically, whole brain radiation therapy (WBRT) was the main treatment modality used for patients with BM, and the median survival of untreated patients was one month [1]. However, improvements in surgery and the extensive use of new technologies for local treatment, as well as improvement in systemic therapies, has led to the reconsideration of treatment strategies, by taking into account the established prognostic factors and by selecting subgroups of long-term survivors for whom intracranial control has become the major endpoint of treatment and, thus, for whom WBRT alone is no longer suitable.

In addition to these prognostic factors, due to different natural histories and responses to treatment according to the histological type and molecular characteristics of the primary tumor, the primary site has become a determining factor in the therapeutic decision. Indeed, diagnosis-specific classifications have highlighted the importance of the primary site in determining differences in prognostic factors for survival, as well as in median survival (median survival: 25 and 13–17 months for graded prognostic assessment (GPA) 4 breast cancer (BC) and other primaries, respectively) [2].

However, international guidelines are based on randomized controlled studies involving no more than 10% of patients with BM from BC [3,4]. In addition, there are no randomized controlled trials conducted in patients with BM from BC. As findings from diagnosis-specific classifications are basically different according to the primary site [2], it could be hypothesized that results from the above-mentioned randomized controlled trials (RCTs) would have been different in a population composed exclusively of patients with BM from BC. Therefore, this review focuses on evidence-based medicine for the use of WBRT in the management of BM from BC and will discuss the application of international guidelines for BM management in patients with BC, particularly related to WBRT.

## 2. Results

Therapeutic modalities for BM include surgery, stereotactic radiosurgery (SRS), WBRT, systemic therapies or a combination of these methods.

### 2.1. Whole Brain Radiation Therapy (WBRT) Alone

It was shown that WBRT, compared to the best supportive care for BM, slightly improved median overall survival (3–6 months *vs.* 1 month, respectively) [1,5]. A complete or partial response and an improvement in neurological symptoms were reached in 30%–60% of cases, with variable definitions of these criteria among the studies [6]. Two studies assessed prognostic factors associated with WBRT response [7,8] and found BM volume to be the most important factor and not BM number. In addition, Nieder *et al.* [7] found that patients with BC (as with those with non-small cell lung cancer) were more likely to present a sustainable response to WBRT compared to those with other histologies. Several authors have retrospectively investigated the response rate to WBRT in patients with BM from BC (mostly multiple BM) and reported a 65%–82% response rate [7,9] with a recurrence rate between 0% and 50% (Table 1) [7,10].

### 2.2. Protraction

In order to improve brain control and survival while minimizing delayed side effects, many studies have compared different radiation therapy schemes used on patients with mixed primaries [6,12–15]. No difference in either the overall survival rate or acute toxicity was observed among the various fractionation schemes studied. However, none of these trials reported data related to intracerebral progression-free survival, tumor response rate or quality of life based on a validated questionnaire. Nonetheless, data from retrospective studies [16,17] seem to favor schemes with lower doses per fraction, which would evidently result in fewer late neurocognitive side effects. The results of one retrospective study [18] comparing two fractionation schemes for BM from BC led to the same conclusions. Usual schemes range from 30 Gy/10 fractions to 40 Gy/20 fractions irrespective of the primary malignancy.

### 2.3. WBRT and Radiosensitizers

Four RCTs (including more than 20 patients with BM from BC) and one meta-analysis involving 2217 patients tested the association of WBRT with several radiosensitizers. Results showed a greater incidence of toxicity without any benefit in terms of local control or survival (Table 2) [19–22].

The use of efaproxiral generated interest in a subgroup of patients with BM from BC. In an RCT by Suh *et al.*, the significant impact of efaproxiral was observed on the median survival (which was doubled) of pre-stratified patients with BC, and there was an increase of 13% in terms of tumor response for the population with lung/breast (*p* = 0.01) [21,23]. Subsequent to these encouraging results, the authors conducted an RCT assessing efaproxiral in the treatment of BM from BC (ENRICH trial: Enhancing WBRT In patients with BC and hypoxic BM). However, the results of this trial in which 365 patients were enrolled, most of whom had two brain lesions, were negative [24].

### 2.4. Radio-Chemotherapy

Although chemotherapy has traditionally played a limited role in the treatment of BM, predominantly due to its low potency to cross the blood brain barrier, it has been the subject of several studies for use in combination with WBRT, mainly for BM from lung cancer. However, only two RCTs comparing WBRT with or without concurrent chemotherapy (temozolomide) included patients with primary BC, and this population was widely underrepresented (Table 3) [25,26].

These trials showed that at the best, there was an improvement in local control and an increased time to cerebral progression. However, this was at the cost of increased toxicity, and there was no improvement in survival.

At the San Antonio Breast Cancer Symposium, 2011, Kirova *et al.* reported the results of a randomized phase II trial assessing WBRT with or without temozolomide as a radiosensitizer for patients with BM from BC [27]; no advantage in tumor control was found using the addition of temozolomide.

Further trials assessing chemotherapy combined with targeted therapies and WBRT are currently under investigation.

### 2.5. WBRT and Targeted Therapies

Therapies targeting the human epidermal growth factor receptor 2 (HER2) have been evaluated or are currently under investigation in combination with WBRT for BMBC.

Only one retrospective study on 31 patients showed the feasibility of the trastuzumab-WBRT combination with good tolerance and an encouraging objective response [28]. A recently completed open-label, non-randomized, multicenter trial has investigated the safety and efficacy of the trastuzumab-WBRT combination (NCT01363986) and should provide more information.

The use of lapatinib concurrently with WBRT has been assessed in a feasibility phase I study for patients with BM from HER2-positive BC, but this study did not meet the predefined criteria for feasibility (upper bound of the dose-limiting toxicity: 95%; confidence interval: <30%) [29]. However, the combination of lapatinib with WBRT is currently being examined in two ongoing phase II studies targeting patients with BM from primary lung or BC, with the main objective of evaluating the response rate (NCT01218529, NCT01622868).

Finally, an ongoing feasibility phase I study is currently examining different sequences of combined trastuzumab emtansine (TDM-1) and WBRT in patients with BM from HER2-positive BC (brain radiation and TDM-1 in HER2-positive metastatic BC: BIRTH trial).

### 2.6. Role of a Boost Added to WBRT

The benefit of an SRS-boost as part of WBRT has been shown in patients with oligometastatic BM [30–32]. However, for multiple BM (>3), the addition of a boost did not lead to better results [15].

The Radiation Therapy Oncology Group (RTOG)-9508 trial (comprising only 10% of patients with BM from BC) showed that WBRT plus SRS was better than WBRT alone in terms of survival rates for patients with single BM and that there was a trend towards a survival benefit for patients with 2–3 BM [30]. Kondziolka *et al.* conducted an RCT with a similar design, for patients with 2–4 BM [31]. The sample size was determined to detect a difference in the primary outcome measure of local control after radiosurgery plus WBRT, and only 27 patients were randomized. No survival advantage of a boost was noted, even though there was a trend towards longer survival in the boost arm, an increased time to progression (36 *vs.* 6 months, *p* = 0.0005) and a dramatic increase in the rate of one-year local control (92% *vs.* 8%, *p* = 0.0016). In a meta-analysis, involving 358 patients with 1–4 BM from unselected primary tumors, no significant difference was observed in the survival between the patients with or without a boost (*HR* = 0.82, 95% CI 0.65; 1.02) [32]. However, in the WBRT + SRS arms, patients with single BM had a significantly increased median overall survival (6.5 *vs.* 4.9 months, *p* = 0.04) and less local recurrence (*HR* = 0.27, 95% CI 0.14; 0.52) than the patients receiving only WBRT. In addition, a statistically significant improvement in Karnofsky performance status (KPS) and a decreased use of corticosteroids was observed. To our knowledge, there is no specific study for BM from BC in this context, but these data promote an additional SRS-boost for patients with 1–3 BM. However, if extra-cerebral disease is uncontrolled, giving a short life expectancy, the value of maximalist intra-cerebral control, and thus of a boost, remains questionable.

Four ongoing prospective trials are currently assessing the interest in, or feasibility of, an integrated boost during WBRT (with or without hippocampal sparing) for patients with multiple BMs from mixed primaries (NCT00876759; NCT01414738; NCT01218542; and NCT01046123).

### 2.7. Role of WBRT after Radiosurgery

The value of WBRT in addition to an SRS procedure was the subject of two randomized phase III trials [33,34]. However, in these two trials, only 10% of the included population were patients with BM from BC. The study by Aoyama *et al.* [33] randomized 132 patients with a KPS ≥70 and 1–4 BM, to receive either SRS alone or SRS plus WBRT. At one year, patients receiving the combination had better local control (initial metastatic site) (88.7% *vs.* 72.5%, *p* = 0.002), better remote control in the brain (58.5% *vs.* 36.3% *p* = 0.003) and one-year actuarial survival not being significantly improved (38.5% *vs.* 28.4%, *p* = 0.42), but the median survival was similar. There was no difference in the neurological death rate and or incidence of toxicity (although, neurocognitive evaluations were lacking) between the two arms. The authors concluded that omission of WBRT was appropriate. This study was the basis of a second analysis focusing on mini mental state examination (MMSE) change as a function of the treatment arm [35]. The MMSE score was available for 110 out of the 132 enrolled patients. For patients with a baseline or post-treatment MMSE ≥27, the median time to MMSE decline was significantly longer in the combined therapy arm (16.5 *vs.* 7.6 months, *p* = 0.05), suggesting that the recurrence itself may be responsible for neurocognitive deterioration.

Similar results were observed in the European Organization for Research and Treatment of Cancer (EORTC) trial, which included 199 patients with 1–3 BM who were randomized to SRS alone or SRS plus WBRT [34]. Upfront WBRT led to a significant decrease in brain failure rate (initial site: 19% *vs.* 31%, *p* = 0.04; remote site: 33% *vs.* 48%, *p* = 0.023) and in neurological deaths, without any difference in functional independence time or survival. This study generated a second analysis reporting the quality of life and a self-reporting assessment of cognitive function [36]. A statistically significant and clinically meaningful difference in global health-related quality of life mean scores was detected at nine months in favor of patients who had observation alone, but this difference was transient and no longer observed at 12 months. However, the mean difference was statistically significant for cognitive functioning at 12 months (mean, 80.4; SE, 3.7 for observation *vs.* mean, 69.7; SE, 4.0 for WBRT; *p* = 0.0486).

Finally, a retrospective trial suggested a survival advantage for patients with single BM and controlled extra-cranial disease who underwent SRS followed by WBRT, compared with those receiving only SRS [37].

The above is a summary of the literature related to the current debate regarding the best choice for patients with oligometastatic brain disease. On the one hand, since WBRT appears to significantly reduce the number of recurrent BMs, it could reasonably be concluded that the trial from Aoyama *et al.* [33] supports the use of upfront WBRT in the treatment of 1–4 BM; but on the other hand, opponents [34] argue that WBRT can be omitted, since it has no impact on survival, and recurrence might be successfully treated with salvage therapy, thus avoiding any neurotoxic effect in patients without relapse.

An ongoing multicenter randomized phase III trial with the primary end-point of overall survival is assessing the neurocognitive effect of WBRT added to SRS for patients with 1–3 BM from unselected solid tumors as a secondary end-point (NCT00377156), in order to verify the results of the RCT from Chang *et al.* [38], which found that patients in the combined arm were significantly more impaired in one neurocognitive test at four months (52% *vs.* 24%). These interesting results required further investigations, as several biases were highlighted (such as the suboptimal time to neurocognitive assessment, a surprisingly high death rate in the combined arm, an imbalance in prognostic factors, baseline neurocognition and tumor volume).

### 2.8. WBRT ± Surgery

Surgery for BM has usually been considered only in cases of vital threat, major neurological risk or the need for histological diagnosis. For patients with a single BM, the treatment strategy has now been modified, based on the results of three RCTs [39–41]. In these trials, patients with a single BM were randomly assigned to receive either WBRT alone or surgery followed by WBRT (Table 4).

These trials included a limited number of patients with BM from BC, but are the only trials to provide information related to this type of therapeutic strategy. Two of the trials showed an improvement in the median survival for patients with controlled extra-cranial metastases who underwent surgery, as well as a significant improvement in functionality (KPS ≥ 70: 38 *vs.* 8 weeks) [39,40,42]. The survival advantage was attributed to a decrease in neurological deaths, and this was not offset by systemic deaths when extra-cranial disease was controlled [39]. However, the third trial of Mintz *et al.* [41] did not show any survival advantage, but the fact that 73% of included patients had uncontrolled extra-cranial disease may explain this result. In addition, in this study, no patient received a cerebral MRI, making questionable the “single” brain metastasis definition, whereas all patients in the Patchell studies were actually assessed by MRI-scans.

### 2.9. Surgery ± WBRT

Surgery without upfront WBRT has also been evaluated in two RCTs, wherein 10% of the patients had BM from BC [34,43]. In these two trials, upfront WBRT led to a lower incidence of brain failure (initial site 10%–27% *vs.* 46%–59%; remote sites 14%–23% *vs.* 37%–42%) and neurological death (14%–28% *vs.* 44% without surgery), without any difference in overall survival. It could be expected that for patients with controlled extra-cranial disease, a survival advantage would emerge from the combined strategy. Less than half of the participating patients in both trials had controlled extra-cranial disease, and no subgroup analysis for survival was performed. Moreover, since the neurological death rate was significantly higher for patients with delayed WBRT in both studies, it appears that salvage therapy is less effective on brain control than upfront WBRT.

Furthermore, two retrospective studies that included patients with single BM and no extra-cerebral disease showed a survival advantage for the group of patients receiving WBRT after BM surgical resection [44,45].

Other therapeutic strategies are currently under investigation, but have not yet been published in RCT, e.g., “on demand” SRS, surgery followed by tumor bed irradiation.

## 3. Discussion

To summarize, scarce data exists in relation to the management of BM, specifically from BC, the purpose of which ranges from maximal brain disease control to symptom palliation. It is considered that, despite a relatively good response rate and good symptom palliation, WBRT alone remains insufficient in the treatment of oligometastatic brain disease and should be considered only for multiple (>4) BMs.

The main dilemma relies on the use of WBRT after local treatment for oligometastatic brain disease (1–4 BM). Despite the benefits that would be delivered in terms of increased brain control, postponing WBRT after local treatment is considered to be a reasonable option, due to the lack of survival benefit from upfront WBRT, as has been discussed in the EORTC trial [34].

However, it is of note that, firstly, some data support the hypothesis that survival after a diagnosis of BM in patients with BC improves with the addition of modern multimodalities treatment [46]; and secondly, it has been suggested that contrary to the effects in patients with BM from other solid tumors, half of the patients with BM from BC can die from neurological causes [47,48].

Participants in the EORTC trial consisted of 53% with BM from lung cancer, 12% with BM from BC and 35% with BM from other causes. Despite a statistically significant decrease in neurological deaths in patients who received upfront WBRT, the median survival was not altered. Death was considered as being from neurological causes if intracranial failure was a component of the cause of death. In other words, the death rate was the same in both arms, whatever the neurological status, meaning that the cause of death was not related to the brain disease, but rather to systemic disease. That is considered to be the reason why an improvement in brain control does not lead to prolonged survival. For patients whose lives are not jeopardized by uncontrolled systemic or primary disease, an improvement in brain control may lead to an increase in median survival, as suggested by some authors [37,44,45]. In the EORTC trial, the cerebral progression rate was 78% in the observation arm *vs.* 48% in the adjuvant WBRT arm (*p* = 0.002) [34]. Hence, it is reasonable to wonder whether the control of brain disease in patients with controlled extra-cranial disease might actually impact on their overall survival. Since the median survival of patients with BM from BC is proven to have doubled compared to other primaries, most likely due to the prolonged efficiency of systemic treatments [2], it seems crucial to reappraise the actual role of adjuvant WBRT in this population. For all the reasons specified here, the results of the BM RCTs (including mainly non-small cell lung cancers) should not be standardized for all histologies.

Moreover, leptomeningeal disease (LMD), which is a well-known pattern of central nervous system (CNS) failure in patients with metastatic breast cancer, is usually associated with a very poor outcome. This failure pattern has been significantly associated with BC as the primary site in patients treated with SRS alone, and WBRT has been found to significantly lower this risk [49,50]. The one-year cumulative incidence of LMD has been estimated to be as high as 24% (95% CI, 9%–41%) for BC compared to 9% (95% CI, 5%–14%) for patients with a non-breast histology (*p* = 0.004) [50].

Some BC subtypes, such as HER2 and triple negative (HER2 negative and hormonal receptors negative), are at high risk of brain recurrence even after initial treatment [51,52], with the former more likely to have controlled extra-cranial disease, making brain control a major issue.

Based on these findings, there are at least two arguments favoring upfront WBRT for oligometastatic brain disease from BC: firstly, the fact that patients with BC are more likely to have controlled systemic disease and are thus more exposed to suffering from neurological death; and secondly, the fact that leptomeningeal disease (which is a typical pattern of CNS failure in patients with BC) is associated with a very poor prognosis and quality of life, but its incidence is lowered by WBRT.

Opponents to upfront WBRT propose an “on demand” SRS strategy, in order to avoid potential neurotoxicity, with an MRI survey every three months. However, it is also known that brain recurrence negatively impacts neurocognitive function [35,53]. Furthermore, in some cases, after multiple SRS sessions, WBRT will be no longer possible due to the cumulative radiation toxicity risk. Finally, brain recurrence is not always accessible to SRS, and if so, WBRT will be mandatory, with less efficiency, due to the BM size; therefore, neurological deterioration could occur earlier than radio-induced toxicity.

The problem with upfront WBRT concerns long-term neurotoxicities as assessed through RCTs [54]. The ideal strategy for oligometastatic brain disease would be a local treatment followed by a preventive systemic treatment, which still needs to be defined. Efforts are currently being developed to minimize WBRT neurotoxicity, while providing maximal brain control. Hence, the Radiation Therapy Oncology Group (RTOG) has recently reported the effectiveness of memantine (*N*-methyl-d-aspartate receptor antagonist) in preventing cognitive dysfunction following WBRT [55]; hippocampal sparing during WBRT is currently under investigation in three RCTs (NCT01227954, NCT01414738 and NCT01942980) with encouraging results emerging from a phase II trial from the RTOG presented at the American Society for Radiation Oncology (ASTRO) meeting 2013.

## 4. Materials and Method

Relevant studies were identified by searching the electronic database, Medlin/PubMed (National Library of Medicine, Bethesda, MA, USA), between January 2000 and September 2013, using the following keywords: WBRT, BM, BC. Reference lists from these sources were then manually searched to identify additional relevant publications. To be eligible for inclusion, studies had to meet the following criteria: published data on WBRT alone or combined with other treatments, in the management of newly diagnosed BM, either in the particular setting of BM from BC or in a general setting from which data for patients with primary BC (more than 20) were extracted and included in the study. As this review focuses on the efficiency of WBRT, studies on radiation therapy schedules and treatments combined with WBRT, such as local (surgery, radiosurgery and radiation boost), radiosensitizers, chemotherapy and targeted therapies, were also included. Articles were excluded from our review if they were non-English language papers, individual case reports, review articles, phase I and II trials for which phase III trials were already available, trials not assessing WBRT itself and studies not including patients with BM from BC. Data extracted from the studies were: the number of patients accrued and/or evaluated, the impact of therapeutic combinations and radiation therapy schedules on the response rate to treatments, overall survival and toxicity.

The aim was to evaluate whether WBRT for patients with BM from BC is still considered to be an appropriate treatment.

## 5. Conclusions

When the benefit of controlling brain disease is not offset by active extra-cranial disease, there is an actual need for the use of aggressive treatments in patients with BM with controlled systemic disease. Due to the improvement of systemic disease treatment in BC, the overall survival of these patients has been significantly lengthened. Some particular molecular profiles of BC, such as HER2-positive and triple negative, are responsible for an increased incidence of BM. In these patients the survival rate is actually associated with neurological death, making BM treatment in this population an actual challenge. Indeed, brain disease control in this specific population is crucial and potentially could impact overall survival. For these reasons, the findings of the EORTC phase III study should not be generalized to a particular population, and the upfront WBRT should be reappraised focusing on the impact of brain tumor control on overall survival.

## Figures and Tables

**Table 1.** Studies assessing Whole Brain Radiation Therapy (WBRT) alone in patients with brain metastases (BM) from breast cancer (BC).

Study	Ref.	*N*	BM number	Radiation schedule	Response rate * (%)	Recurrence rate (%)
Nieder *et al.*, 1997	[7]	46	Median:4	30 Gy/10 f	65 a	0
Ogura *et al.*, 2003	[9]	36	Multiple	−30 Gy/10 f −50 Gy/25 f Boost: 10 pts	82 b	32
Mahmoud-Ahmed *et al.*, 2002	[10]	116	Single:20 2–3:38 4–9:50 >9:8	30 Gy/10 f	NR	50
Le Scodan *et al.*, 2007	[11]	117	Multiple	30 Gy/10 f	NR	42.5

Abbreviations: *N*, number of patients; pts, patients; WBRT, whole brain radiation therapy; BM, brain metastases; BC, breast cancer; f, fraction; NR, not reported;

* Volumetric response was defined as the following:

a complete remission = complete disappearance of a contrast-enhancing lesion on CT-scan; partial remission = volume reduction of >50%; progression = volume increase of >25%; the remaining were scored as no change; and

b not defined.

**Table 2.** Radiosensitizers + WBRT *vs.* WBRT: randomized studies, including patients with BM from BC.

Study	Ref.	Randomization arm	*N*	Primary (%)	Median survival (months)	6-Month survival	Response Rate
Komarnicky *et al.*, 1991	[19]	30 Gy/6 fr + misonidazole	196	L: NR B: NR O: NR	3.1	68	NR
		30 Gy/6 fr	200		4.1	83	
		30 Gy/10 fr + misonidazole	190		3.9	65	
		30 Gy/10 fr	193		4.5	72	

Mehta *et al.*, 2003	[20]	30 Gy/10 fr + MGd	193	L: 62%	5.1	82	NR
				B: 19%			
		30 Gy/10 fr	208	O: 19%	5.8	85	
					*p* = 0.68		

Suh *et al.*, 2006	[21]	30 Gy/10 fr + O_2_ + efaproxiral	265	L: 56%	5.4	NR	46
				B: 21%			38
		30 Gy/10 fr + O_2_	250	O: 23%	4.4		*p* = 0.1
					*p* = 0.16		

Knisely *et al.*, 2008	[22]	37.5 Gy/15 fr + thalidomide	84	L: 62%	3.9	26%	NR
				B: 18%			
		37.5 Gy/15 fr	92	O: 20%	3.9	28%	
						*p* = 0.88	

Abbreviations: *N*, number of patients; WBRT, whole brain radiation therapy; fr, fractions; L, lung; B, breast; O, other; NR, not reported; MGd, motexafin gadolinium.

**Table 3.** Randomized studies (including patients with BM from BC) assessing WBRT + chemotherapy *vs.* WBRT alone.

Study	Ref.	Randomization arm	*N*	Primary	Median survival (months)	Response rate
Antonadou *et al.*, 2002	[25]	40 Gy/20 fr	23	L: 83%	7.0	67%
				B: 11%		
		40 Gy/20 fr + TMZ	25	O: 6%	8.6 NS	96% (*p* = 0.017)

Verger *et al.*, 2005	[26]	30 Gy/10 fr	41	L: 51%	3.1	32%
				B: 16%		
		30 Gy/10 fr + TMZ	41	O: 33%	4.5 NS	32% (*p* = NS)

Abbreviations: *N*, number of patients; TMZ, temozolomide; L, lung; B, breast; O, other; NS, non-significant.

**Table 4.** WBRT *vs.* surgery + WBRT: randomized trials.

Study	Ref.	BM treatment	Radiation schedule	Patients number	Median survival (months)	*p*
Patchell *et al.*, 1990	[39]	Biopsy + WBRT	36 Gy/12 fr	23	3.4	<0.01
		Surgery + WBRT		25	9.2	

Vecht *et al.*, 1993	[40]	WBRT	40 Gy/20 BID	31	6	0.04
		Surgery + WBRT		32	10	

Mintz *et al.*, 1996	[41]	WBRT	30 Gy/10 fr	43	6.3	0.24 (NS)
		Surgery + WBRT		41	5.6	

Abbreviations: WBRT, whole brain radiation therapy; BM, brain metastases; fr, fractions; BID, twice fractions daily; and NS, non-significant.

## References

[1] Zimm S., Wampler G.L., Stablein D., Hazra T., Young H.F. (1981). Intracerebral metastases in solid-tumor patients: Natural history and results of treatment. Cancer.

[2] Sperduto P.W., Chao S.T., Sneed P.K., Luo X., Suh J., Roberge D., Bhatt A., Jensen A.W., Brown P.D., Shih H. (2010). Diagnosis-specific prognostic factors, indexes, and treatment outcomes for patients with newly diagnosed brain metastases: A multi-institutional analysis of 4259 patients. Int. J. Radiat. Oncol. Biol. Phys.

[3] Bhangoo S.S., Linskey M.E., Kalkanis S.N. (2011). Evidence-based guidelines for the management of brain metastases. Neurosurg. Clin. N. Am.

[4] Gaspar L.E., Mehta M.P., Patchell R.A., Burri S.H., Robinson P.D., Morris R.E., Ammirati M., Andrews D.W., Asher A.L., Cobbs C.S. (2010). The role of whole brain radiation therapy in the management of newly diagnosed brain metastases: A systematic review and evidence-based clinical practice guideline. J. Neurooncol.

[5] Horton J., Baxter D.H., Olson K.B. (1971). The management of metastases to the brain by irradiation and corticosteroids. Am. J. Roentgenol. Radium Ther. Nucl. Med.

[6] Borgelt B., Gelber R., Kramer S., Brady L.W., Chang C.H., Davis L.W., Perez C.A., Hendrickson F.R. (1980). The palliation of brain metastases: Final results of the first two studies by the Radiation Therapy Oncology Group. Int. J. Radiat. Oncol. Biol. Phys.

[7] Nieder C., Berberich W., Schnabel K. (1997). Tumor-related prognostic factors for remission of brain metastases after radiotherapy. Int. J. Radiat. Oncol. Biol. Phys.

[8] Phillips T.L., Scott C.B., Leibel S.A., Rotman M., Weigensberg I.J. (1995). Results of a randomized comparison of radiotherapy and bromodeoxyurine with radiotherapy alone for brain metastases: Report of RTOG trial 89–05. Int. J. Radiat. Oncol. Biol. Phys.

[9] Ogura M., Mitsumori M., Okumura S., Yamauchi C., Kawamura S., Oya N., Nagata Y., Hiraoka M. (2003). Radiation therapy for brain metastases from breast cancer. Breast Cancer.

[10] Mahmoud-Ahmed A.S., Suh J.H., Lee S.Y., Crownover R.L., Barnett G.H. (2002). Results of whole brain radiotherapy in patients with brain metastases from breast cancer: A retrospective study. Int. J. Radiat. Oncol. Biol. Phys.

[11] Le Scodan R., Massard C., Mouret-Fourme E., Guinebretierre J.M., Cohen-Solal C., de Lalande B., Moisson P., Breton-Callu C., Gardner M., Goupil A. (2007). Brain metastases from breast carcinoma: Validation of the radiation therapy oncology group recursive partitioning analysis classification and proposition of a new prognostic score. Int. J. Radiat. Oncol. Biol. Phys.

[12] Harwood A.R., Simson W.J. (1977). Radiation therapy of cerebral metastases: A randomized prospective clinical trial. Int. J. Radiat. Oncol. Biol. Phys.

[13] Borgelt B., Gelber R., Larson M., Hendrickson F., Griffin T., Roth R. (1981). Ultra-rapid high dose irradiation schedules for the palliation of brain metastases: Final results of the first two studies by the Radiation Therapy Oncology Group. Int. J. Radiat. Oncol. Biol. Phys.

[14] Haie-Meder C., Pellae-Cosset B., Laplanche A., Lagrange J.L., Tuchais C., Nogues C., Arriagada R. (1993). Results of a randomized clinical trial comparing two radiation schedules in the palliative treatment of brain metastases. Radiother. Oncol.

[15] Murray K.J., Scott C., Greenberg H.M., Emami B., Seider M., Vora N.L., Olson C., Whitton A., Movsas B., Curran W. (1997). A randomized phase III study of accelerated hyperfractionation *vs.* standard in patients with unresected brain metastases: A report of the Radiation Therapy Oncology Group (RTOG) 9104. Int. J. Radiat. Oncol. Biol. Phys.

[16] DeAngelis L.M., Delattre J.Y., Posner J.B. (1989). Radiation-induced dementia in patients cured of brain metastases. Neurology.

[17] Crossen J.R., Garwood D., Glatstein E., Neuwelt E.A. (1994). Neurobehavioral sequelae of cranial irradiation in adults: A review of radiation-induced encephalopathy. J. Clin. Oncol.

[18] Rades D., Lohynska R., Veninga T., Stalpers L.J., Schild S.E. (2007). Evaluation of 2 whole-brain radiotherapy schedules and prognostic factors for brain metastases in breast cancer patients. Cancer.

[19] Komarnicky L.T., Phillips T.L., Martz K., Asbell S., Isaacson S., Urtasun R. (1991). A randomized phase III protocol for the evaluation of misonidazole combined with radiation in the treatment of patients with brain metastases (RTOG-7916). Int. J. Radiat. Oncol. Biol. Phys.

[20] Mehta M.P., Rodrigus P., Terhaard C.H., Rao A., Suh J., Roa W., Souhami L., Bezjak A., Leibenhaut M., Komaki R. (2003). Survival and neurologic outcomes in a randomized trial of motexafin gadolinium and whole-brain radiation therapy in brain metastases. J. Clin. Oncol.

[21] Suh J.H., Stea B., Nabid A., Kresl J.J., Fortin A., Mercier J.P., Senzer N., Chang E.L., Boyd A.P., Cagnoni P.J. (2006). Phase III study of efaproxiral as an adjunct to whole-brain radiation therapy for brain metastases. J. Clin. Oncol.

[22] Knisely J.P., Berkey B., Chakravarti A. (2008). A phase III study of conventional radiation therapy plus thalidomide *vs.* conventional radiation therapy for multiple brain metastases (RTOG 0118). Int. J. Radiat. Oncol. Biol. Phys.

[23] Scott C., Suh J., Stea B., Nabid A., Hackman J. (2007). Improved survival, quality of life, and quality-adjusted survival in breast cancer patients treated with efaproxiral (Efaproxyn) plus whole-brain radiation therapy for brain metastases. Am. J. Clin. Oncol.

[24] Suh J.H., Stea B., Tankel K., Marsiglia H., Belkacemi Y., Gomez H., Falcone-Lizaraso S., May J., Saunders M. (2008). Results of the phase III ENRICH (RT-016) study of efaproxiral administered concurrently with whole brain radiation therapy (WBRT) in women with brain metastases from breast cancer. Int. J. Radiat. Oncol. Biol. Phys.

[25] Antonadou D., Paraskevaidis M., Sarris G., Coliarakis N., Economou I., Karageorgis P., Throuvalas N. (2002). Phase II randomized trial of temozolomide and concurrent radiotherapy in patients with brain metastases. J. Clin. Oncol.

[26] Verger E., Gil M., Yaya R., Viñolas N., Villà S., Pujol T., Quintó L., Graus F. (2005). Temozolomide and concomitant whole brain radiotherapy in patients with brain metastases: A phase II randomized trial. Int. J. Radiat. Oncol. Biol. Phys.

[27] Kirova Y.M., Hajage D., Gerber S., le Scodan R., Pierga J.Y., Bourgier C., Campana F., Asselain B., Fourquet A., Levy C. (2012). Whole-brain radiation therapy plus temozolomide for the treatment of brain metastases from breast cancer: A randomized prospective multicenter phase II study. Cancer Res.

[28] Chargari C., Idrissi H.R., Pierga J.Y., Bollet M.A., Diéras V., Campana F., Cottu P., Fourquet A., Kirova Y.M. (2011). Preliminary results of whole brain radiotherapy with concurrent trastuzumab for treatment of brain metastases in breast cancer patients. Int. J. Radiat. Oncol. Biol. Phys.

[29] Lin N.U., Freedman R.A., Ramakrishna N., Younger J., Storniolo A.M., Bellon J.R., Come S.E., Gelman R.S., Harris G.J., Henderson M.A. (2013). A phase I study of lapatinib with whole brain radiotherapy in patients with human epidermal growth factor receptor 2 (HER2)-positive breast cancer brain metastases. Breast Cancer Res. Treat.

[30] Andrews D.W., Scott C.B., Sperduto P.W., Flanders A.E., Gaspar L.E., Schell M.C., Werner-Wasik M., Demas W., Ryu J., Bahary J.P. (2004). Whole brain radiation therapy with or without stereotactic radiosurgery boost for patients with one to three brain metastases: Phase III results of the RTOG 9508 randomised trial. Lancet.

[31] Kondziolka D., Patel A., Lunsford L.D., Kassam A., Flickinger J.C. (1999). Stereotactic radiosurgery plus whole brain radiotherapy *vs.* radiotherapy alone for patients with multiple brain metastases. Int. J. Radiat. Oncol. Biol. Phys.

[32] Patil C.G., Pricola K., Garg S.K., Bryant A., Black K.L. (2010). Whole brain radiation therapy (WBRT) alone *vs.* WBRT and radiosurgery for the treatment of brain metastases. Cochrane Database Syst. Rev.

[33] Aoyama H., Shirato H., Tago M., Nakagawa K., Toyoda T., Hatano K., Kenjyo M., Oya N., Hirota S., Shioura H. (2006). Stereotactic radiosurgery plus whole-brain radiation therapy *vs.* stereotactic radiosurgery alone for treatment of brain metastases: A randomized controlled trial. JAMA.

[34] Kocher M., Soffietti R., Abacioglu U., Villà S., Fauchon F., Baumert B.G., Fariselli L., Tzuk-Shina T., Kortmann R.D., Carrie C. (2011). Adjuvant whole-brain radiotherapy *vs.* observation after radiosurgery or surgical resection of one to three cerebral metastases: Results of the EORTC 22952-26001 study. J. Clin. Oncol.

[35] Aoyama H., Tago M., Kato N., Toyoda T., Kenjyo M., Hirota S., Shioura H., Inomata T., Kunieda E., Hayakawa K. (2007). Neurocognitive function of patients with brain metastasis who received either whole brain radiotherapy plus stereotactic radiosurgery or radiosurgery alone. Int. J. Radiat. Oncol. Biol. Phys.

[36] Soffietti R., Kocher M., Abacioglu U.M., Villà S., Fauchon F., Baumert B.G., Fariselli L., Tzuk-Shina T., Kortmann R.D., Carrie C. (2013). A European Organisation for Research and Treatment of Cancer phase III trial of adjuvant whole-brain radiotherapy *vs.* observation in patients with one to three brain metastases from solid tumors after surgical resection or radiosurgery: Quality-of-life results. J. Clin. Oncol.

[37] Pirzkall A., Debus J., Lohr F., Fuss M., Rhein B., Engenhart-Cabillic R., Wannenmacher M. (1998). Radiosurgery alone or in combination with whole-brain radiotherapy for brain metastases. J. Clin. Oncol.

[38] Chang E.L., Wefel J.S., Hess K.R., Allen P.K., Lang F.F., Kornguth D.G., Arbuckle R.B., Swint J.M., Shiu A.S., Maor M.H. (2009). Neurocognition in patients with brain metastases treated with radiosurgery or radiosurgery plus whole-brain irradiation: A randomised controlled trial. Lancet Oncol.

[39] Patchell R.A., Tibbs P.A., Walsh J.W., Dempsey R.J., Maruyama Y., Kryscio R.J., Markesbery W.R., Macdonald J.S., Young B. (1990). A randomized trial of surgery in the treatment of single metastases to the brain. N. Engl. J. Med.

[40] Vecht C.J., Haaxma-Reiche H., Noordijk E.M., Padberg G.W., Voormolen J.H., Hoekstra F.H., Tans J.T., Lambooij N., Metsaars J.A., Wattendorff A.R. (1993). Treatment of single brain metastasis: Radiotherapy alone or combined with neurosurgery?. Ann. Neurol.

[41] Mintz A.H., Kestle J., Rathbone M.P., Gaspar L., Hugenholtz H., Fisher B., Duncan G., Skingley P., Foster G., Levine M. (1996). A randomized trial to assess the efficacy of surgery in addition to radiotherapy in patients with a single cerebral metastasis. Cancer.

[42] Noordijk E.M., Vecht C.J., Haaxma-Reiche H., Padberg G.W., Voormolen J.H., Hoekstra F.H., Tans J.T., Lambooij N., Metsaars J.A., Wattendorff A.R. (1994). The choice of treatment of single brain metastasis should be based on extracranial tumor activity and age. Int. J. Radiat. Oncol. Biol. Phys.

[43] Patchell R., Tibbs P.A., Regine W.F., Dempsey R.J., Mohiuddin M., Kryscio R.J., Markesbery W.R., Foon K.A., Young B. (1998). Postoperative radiotherapy in the treatment of single metastases to the brain: A randomized trial. JAMA.

[44] Smalley S.R., Schray M.F., Laws E.R., O’Fallon J.R. (1987). Adjuvant radiation therapy after surgical resection of solitary brain metastasis: Association with pattern of failure and survival. Int. J. Radiat. Oncol. Biol. Phys.

[45] DeAngelis L.M., Mandell L.R., Thaler H.T., Kimmel D.W., Galicich J.H., Fuks Z., Posner J.B. (1989). The role of postoperative radiotherapy after resection of single brain metastases. Neurosurgery.

[46] Park B.B., Uhm J.E., Cho E.Y., Choi Y.L., Ji S.H., Nam D.H., Lee J., Park W., Huh S.J., Park Y.H. (2009). Prognostic factor analysis in patients with brain metastases from breast cancer: How can we improve the treatment outcomes?. Cancer Chemother. Pharmacol.

[47] Eichler A.F., Kuter I., Ryan P., Schapira L., Younger J., Henson J.W. (2008). Survival in patients with brain metastases from breast cancer: The importance of HER-2 status. Cancer.

[48] Clayton A.J., Danson S., Jolly S., Ryder W.D., Burt P.A., Stewart A.L., Wilkinson P.M., Welch R.S., Magee B., Wilson G. (2004). Incidence of cerebral metastases in patients treated with trastuzumab for metastatic breast cancer. Br. J. Cancer.

[49] Jo K.I., Lim D.H., Kim S.T., Im Y.S., Kong D.S., Seol H.J., Nam D.H., Lee J.I. (2012). Leptomeningeal seeding in patients with brain metastases treated by gamma knife radiosurgery. J. Neurooncol.

[50] Atalar B., Modlin L.A., Choi C.Y., Adler J.R., Gibbs I.C., Chang S.D., Harsh G.R., Li G., Nagpal S., Hanlon A. (2013). Risk of leptomeningeal disease in patients treated with stereotactic radiosurgery targeting the postoperative resection cavity for brain metastases. Int. J. Radiat. Oncol. Biol. Phys.

[51] Karam I., Hamilton S., Nichol A., Woods R., Speers C., Kennecke H., Tyldesley S. (2013). Population-based outcomes after brain radiotherapy in patients with brain metastases from breast cancer in the pre-Trastuzumab and Trastuzumab eras. Radiat. Oncol.

[52] Vern-Gross T.Z., Lawrence J.A., Case L.D., McMullen K.P., Bourland J.D., Metheny-Barlow L.J., Ellis T.L., Tatter S.B., Shaw E.G., Urbanic J.J. (2012). Breast cancer subtype affects patterns of failure of brain metastases after treatment with stereotactic radiosurgery. J. Neurooncol.

[53] Li J., Bentzen S.M., Renschler M., Mehta M.P. (2007). Regression after whole brain radiation therapy for brain metastases correlates with survival and improved neurocognitive function. J. Clin. Oncol.

[54] Tallet A.V., Azria D., Barlesi F., Spano J.P., Carpentier A.F., Gonçalves A., Metellus P. (2012). Neurocognitive function impairment after whole brain radiotherapy for brain metastases: Actual assessment. Radiat. Oncol.

[55] Brown P.D., Pugh S., Laack N.N., Wefel J.S., Khuntia D., Meyers C., Choucair A., Fox S., Suh J.H., Roberge D. (2013). Memantine for the prevention of cognitive dysfunction in patients receiving whole-brain radiotherapy: A randomized, double-blind, placebo-controlled trial. Neuro Oncol.

